# Silicification of Root Tissues

**DOI:** 10.3390/plants9010111

**Published:** 2020-01-15

**Authors:** Alexander Lux, Zuzana Lukačová, Marek Vaculík, Renáta Švubová, Jana Kohanová, Milan Soukup, Michal Martinka, Boris Bokor

**Affiliations:** 1 Department of Plant Physiology, Faculty of Natural Sciences, Comenius University in Bratislava, 842 15 Bratislava, Slovakia; alexander.lux@uniba.sk (A.L.); zuzana.lukacova@uniba.sk (Z.L.); marek.vaculik@uniba.sk (M.V.); renata.svubova@uniba.sk (R.Š.); jana.kohanova@uniba.sk (J.K.); soukup.em@gmail.com (M.S.); michal.martinka@uniba.sk (M.M.); 2 Institute of Chemistry, Slovak Academy of Sciences, 845 36 Bratislava, Slovakia; 3 Institute of Botany, Plant Science and Biodiversity Centre, Slovak Academy of Sciences, 845 23 Bratislava, Slovakia; 4 Comenius University Science Park, 841 04 Bratislava, Slovakia

**Keywords:** abiotic and biotic stress, cell wall, endodermis, phytoliths, root, silicon, silicon transporters

## Abstract

Silicon (Si) is not considered an essential element, however, its tissue concentration can exceed that of many essential elements in several evolutionary distant plant species. Roots take up Si using Si transporters and then translocate it to aboveground organs. In some plant species, root tissues are also places where a high accumulation of Si can be found. Three basic modes of Si deposition in roots have been identified so far: (1) impregnation of endodermal cell walls (e.g., in cereals, such as *Triticum* (wheat)); (2) formation of Si-aggregates associated with endodermal cell walls (in the Andropogoneae family, which includes *Sorghum* and *Saccharum* (sugarcane)); (3) formation of Si aggregates in “stegmata” cells, which form a sheath around sclerenchyma fibers e.g., in some palm species (*Phoenix* (date palm)). In addition to these three major and most studied modes of Si deposition in roots, there are also less-known locations, such as deposits in xylem cells and intercellular deposits. In our research, the ontogenesis of individual root cells that accumulate Si is discussed. The documented and expected roles of Si deposition in the root is outlined mostly as a reaction of plants to abiotic and biotic stresses.

## 1. Introduction

All roots interact with their environment, which can vary from aquatic to humid air, and in most species with the soil. In all these cases, the root encounters different conditions for its main function—uptake and transport of water and nutrients to the entire plant. The nutrients required for plant growth and development are taken up together with many other substances, some of them being toxic or in toxic concentrations, and some of them absorbed accidentally. For many years, silicon has been considered one of these elements, since it is absorbed and translocated in low concentrations together with water. Practical experiences have been, however, different [1]. In Asian countries, mainly in Japan and China, especially in areas of continuous cultivation of rice, farmers empirically discovered that something was lacking in the soil. For a better rice yield, they began adding ash from rice straw as a supplement to usual fertilizers, such as manure. In fact, Si accumulators, such as rice or sugarcane can remove up to 500 kg Si ha^−1^ per year [2,3] from soil. Intensive farming can thus lead to soil Si depletion and to a deficient harvest of crops [4]. Plants which accumulate Si in significant amounts in these countries offer a good source of Si in their bodies that are not used by farmers, however, e.g., rice hull is often wasted in many countries [5]. Rice hull ash collected by rice farmers, who use rice hull as a source of energy for domestic cooking, contains up to 70% of amorphous SiO_2_. In addition, rice straw is not useful for animals as fodder because of its high Si content and low digestibility [6]. A similar problem of hay with little value can also be found in many countries of Africa.

The situation of marginal interest in Si for plants has gradually changed. The turning points in this research are represented by reviews published several decades ago [7,8]. Recently, many researchers and farmers have realized the usefulness of Si for the cultivation of various crop species [9,10,11,12,13,14]. The International Society for Silicon in Agriculture (ISSAG; http://www.issag.org/home.html) was established and new data have steadily appeared. Apart from the role of Si for optimal plant growth, there are several other research areas where silica deposits are studied and used. Those areas are mainly the fields of palaeontology and archaeology (e.g., in [15,16]). Most of the research is, however, focused on aboveground plant parts—leaves, stems, flower, and fruits. The role of root silicification and places of silica accumulation are much less studied in subterranean plant parts and particularly in roots. In this contribution, we attempt to bring an overview to the readers of the knowledge about the historical and ongoing study of root silica accumulation and its role for the root and entire plant. This may also indicate the gaps in the knowledge, as well as the direction of this important field for future root and plant research.

## 2. History and Recent Knowledge about Si Deposits in Roots

Even though high Si concentrations in plants were confirmed and Si was recognized as one of the 15 elements required for plant growth and development at the beginning of the 20^th^ century [8,17], it is still not considered an essential element of higher plants according to the classic understanding of its essentiality that was proposed many decades ago [18]. This definition has been reconsidered several times because many researchers have confirmed the beneficial effects of Si. This is especially true in grasses and among them rice or sugarcane are known for their requirement of Si to produce healthy and highly productive plants [4,19]. To date, the essentiality of Si has been confirmed only for diatoms and members of the yellow-brown or golden algae [20], as well as for representatives of the Equisetaceae family [21]. The non-essentiality of Si de facto has not been proven [8]. This is likely due to the difficulty of completely removing it from nutrient solutions and obtaining “pure” Si-free plants. Silicic acid absorbed by root cells is deposited and stored in plant tissues, and converted into hard polymerized silica gel [22,23].

Research that deals with the topic of Si formation in the plant body dates back to the beginning of the 19^th^ century. Investigation of silica in the epidermis was performed in several species, including *Triticum*, *Avena*, *Arundo*, and *Equisetum*. In the following decades, several authors described silica bodies in different cells, mostly in the aboveground part of the plant (e.g., in [24,25]. The presence of Si in living cells was confirmed in 1861 [26]. The first classification system for phytoliths was also developed [27]. Between 1895 and 1940, structures of phytoliths in plants gained wider attention, and research on the formation mechanism, taxonomy, and morphology increased and many papers were published with detailed drawings and notations of plant families that produce silica phytoliths, as well as differences in morphology within families (summarized in [28]). In the middle of the 20^th^ century, [29] it was first suggested that Si can probably exist with a combination with galactose in rye and, later, the creation of the cuticle-silica double layer at the leaf surface was documented [30]. The most prevalent form of Si in the shoot is deposited in the form of insoluble polysilicic acid, silica gel [22]. Silica formation was studied extensively in the leaves of many monocotyledonous species, especially grasses, such as rice, maize, barley, and oat [31,32].

Silica deposition in roots was studied in detail after the introduction of analytical electron microscopy in the 1970s and 1980s, and many important data were collected on the silicification of root tissue in grasses [33,34,35]. Deposits of silicon were also observed in the intercellular spaces of the cortical cells near the endodermis in mature roots of *Molinia caerulea* (L.) Moench [36]. At the beginning of silica deposition, deposits took the form of spheres and lined the cavities and afterwards, filled the intercellular spaces. In this study, Si was not detected in the inner tangential walls of the endodermis [36]. However, it was later found that in most of species, Si is closely associated with the inner tangential wall of the root endodermis [33,37,38,39,40,41]. The deposition of Si in the tangential and radial cell walls of the endodermis of rice was confirmed [42]. Several other studies have also described the root endodermis as being the site of Si accumulation in grasses [43]. Yet, Si accumulation has also been observed in the root exodermis and sclerenchyma of rice plants [44,45,46]. The deposition of Si differs between seminal and adventitious roots of some grasses as well. Electron-probe microanalysis also revealed that in the seminal roots, Si was confined to the thickened inner and outer tangential and radial walls of the endodermis in wheat, barley, and oat [40]. Using cryostage analysis, non-polymerized silicon was observed within large metaxylem in the oldest parts of the wheat root. The largest silica deposition was in the endodermis and the leftover soluble silicon was likely transported by the radial walls of some cells of the pericycle and outer parenchyma to the peripheral metaxylem vessels. Afterwards, it was loaded into the large central metaxylem [47]. The adventitious roots of cereals displayed differences in silica deposition. In barley, Si was present in all endodermal cell walls; in oat, Si deposit was located only in the inner tangential and radial walls. In wheat, Si was present or absent in the thickened endodermis, depending on the cultivar. Deposition of silica in roots differs also between accumulators of Si (rice or maize) and Si non-accumulators (onion) [48]. In rice roots, the highest deposition of Si is in the exodermis and it gradually decreases toward the central cylinder [48]. However, it may not be always so, in different rice cultivars and various root types the pattern can be different (AL personal observation). In maize, the Si deposition occurs mainly in the endodermis and root cortex including exodermis shows decreased Si accumulation. In Si non-accumulator onion plants, Si abundance was highest in the exodermis, while it is clearly lower in the root cortex and endodermis [48]. Silica aggregation in non-lignified spots in the cell walls of the endodermis was also described [40] (discussed further in the next section). The changes in suberin lamellae deposition in the root endodermis due to Si was also observed in various plant species [48,49,50,51].

Other specific types of intracellular phytoliths are present in stegmata cells that contain silica. They were observed in roots, however, they were more abundant in the leaves and stems of many plant species, such as the Musaceae family [52] or palms [53]. Recently, stegmata were observed on the outer surface of the sclerenchyma bundles or associated with the sclerenchyma of vascular bundles [54]. The molecular mechanisms of Si uptake in date palm roots was also revealed [54]. Stegmata may be present in the roots of a few orchid species as well [55,56].

In general, the knowledge about root silica deposits in dicotyledonous and gymnosperm plants is scarce. Considerable amounts of silicon are deposited in the root cortical walls of the aluminum tolerant Norway spruce (*Picea abies* L. Karst.) [57]. In the 1990s, there were unconfirmed data about the occurrence of Si deposits in beech [58], however a combination of transmission electron microscopy and electron energy loss spectroscopy confirmed the presence of biogenic silica in beech roots, leaves, in litter, and in the underlying microaggregates of the topsoil [59]. Some silica deposits were observed in the cell walls of beech roots and leaves, whereas pure silica occurred in the root cortical cells as a layer that was closely associated with polyphenolic substances [59]. The important role of fine beech roots was highlighted and only 1% of Si is accumulated in tissues, whereas 99% is returned to the soil mainly through root and leaf decomposition in the form of dissolved Si, which is again taken up by vegetation [60].

## 3. The Root and Its Interaction with Silicon

Silicon is one of the main constituents of the Earth’s crust, where it is found in the form of silicate minerals [61], and soils contain approximately 50%–70% of silicon dioxide [62]. These are exposed to chemical and physical changes in the soil, resulting in the release of Si into the soil. Due to Si abundance in soil, all terrestrial plants contain a certain amount of silicon in their tissues. The Si content on dry matter ranges from 0.1% to 10% [8]. However, the accumulation of Si varies considerably depending on the plant species, which is mainly due to differences in Si uptake by the roots [63]. Roots take up Si in the form of silicic acid (H_4_SiO_4_) when the pH solution is below 9 and the concentration of silicic acid ranges from 0.1 to 0.6 mM in the soil environment [1,8,64]. After that, Si may be accumulated in the root and/or translocated radially into the xylem and deposited in the amorphous form of SiO_2_ in the aboveground plant parts.

Although silica deposition is dominant in the leaves and thus could be joined with the transpiration, there are some crops with Si deposition in the roots. This unique multifunctional plant part is at the forefront of the soil environment. The uniqueness of this organ underlines several specificities, such as its symbiotic interaction with soil microbes and fungi, as well as some anatomical features that cannot be found in the rest of the plant body i.e., the presence of Casparian bands in the endodermis and/or exodermis [65]. 

**Figure 1:**
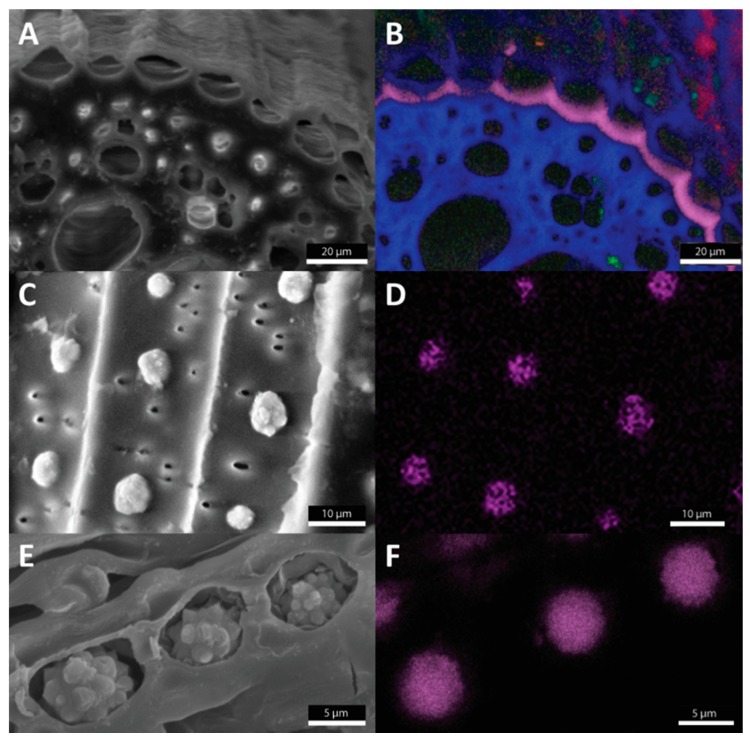
Three modes of silicification in roots investigated by scanning electron microscopy (**A**,**C**,**E**) coupled with X-ray microanalysis (**B**,**D**,**F**), where Si is visualized by pink color. Impregnation of endodermal cell walls is typical for wheat (*Triticum*) (**A**,**B**). Silica aggregates/phytoliths associated with inner tangential cell walls of endodermis are shown in *Sorghum bicolor* (**C**,**D**). Specific stegmata cells filled with silica aggregates/phytoliths occur in *Phoenix dactylifera* (**E**,**F**).

Until now, three types of silica deposition have been identified in roots: I—impregnation of endodermal cell walls, II—silica aggregates/phytoliths in interaction with inner tangential endodermal cell walls, and III—formation of silica aggregates/phytoliths within specific stegmata cells organized on the outer surface of the sclerenchyma fibers (Figure 1).

Several authors have attempted to find the reason and benefits of silica deposition associated with the cell walls in roots. Some of them have even proposed the idea that Si can crosslink the cell wall components [66], giving it the additional strength that is similar to the phenomenon of the lignification process [67]. The metabolic cost of Si deposition is several times less than the lignification process [23]; such increasing mechanical straightness can be energetically more efficient than lignification [68]. On the other hand, silica probably does not provide water-repelling comparable to lignin [41]. The relationship between root silicification and lignification is still to be elucidated [69]. They certainly cannot be considered structural equivalents among other things, since they are completely different in their hydrophobicity. The authors also agree that the formation of silica is on the polymerized lignin, but not on the lignin monomers [69,70,71]. A model of stabilization of negatively charged H_2_SiO_4_ by cell wall lignin has also been suggested [69].

**Figure 2:**
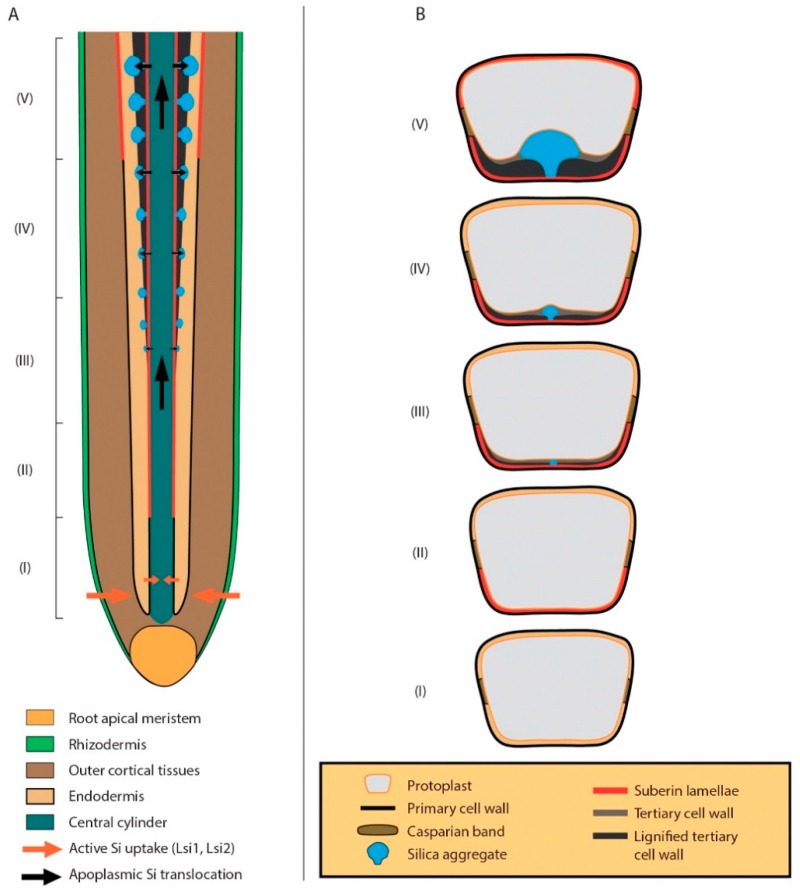
Silica deposition in root endodermis of *Sorghum bicolor* [41,65] modified; with permission from Annals of Botany journal. (**A**) A longitudinal section of root showing transport and deposition of silica in endodermal cells. (**B**) A scheme of silica deposition in a single endodermal cell. I—Endodermal cell with primary cell wall and Casparian strip. II—Suberin lamellae deposition at the inner tangential cell wall. III–IV—Simultaneous deposition of tertiary inner tangential cell wall and the growth of a silica aggregate. Tertiary cell wall grows centripetally as an extending matrix of cellulosic and non-cellulosic polysaccharides that is progressively impregnated by the deposition of various polyphenolic substances polymerizing into lignin. The growth of silica phytolith initiates in a spot predetermined probably by a patchy pattern of lignin polymerization or a local difference in the composition of its constituents. IV—Development of the tertiary cell wall continues and promotes further growth of the silica phytolith. V—The growth of silica phytolith arrests with the end of tertiary cell wall development.

Endodermal silicification associated with the cell wall is arranged in the root according to a specific pattern—it is initiated in the endodermal cells adjacent to the phloem, continues to the xylem poles, and finally, it is observed in the so-called passage cells (Figure 2) [41]. 

The movement of Si is driven by the basipetal water flow, and silica aggregates were observed only in the basal parts of the roots with developed suberin lamellae and lignified tertiary cell walls. The diameters of Si aggregates also correlated with the developmental stage of endodermal cells. In addition, continued silicification could be observed after cell and tissue maturity [72]. The authors also confirmed predetermined places in the cell walls where Si is deposited; there are non-lignified spots where they observed Si in complexes with arabinoxylan-ferulic acid. Arabinoxylans or ferulic acid gives the cell wall of grasses its uniqueness, and according to several other authors, they can be joined with silica deposition [41,73,74,75]. The non-lignified spots in the developed endodermal cell walls can be found in sorghum roots even when the plants are cultivated without Si, which suggests a genetic adaptation of the species. Callose is also important for the silicification of cell walls as was recently found in *Arabidopsis* plants, however this was shown in leaf trichomes [76] and in epidermal papillae and stomata of *Equisetum* [77].

**Figure 3:**
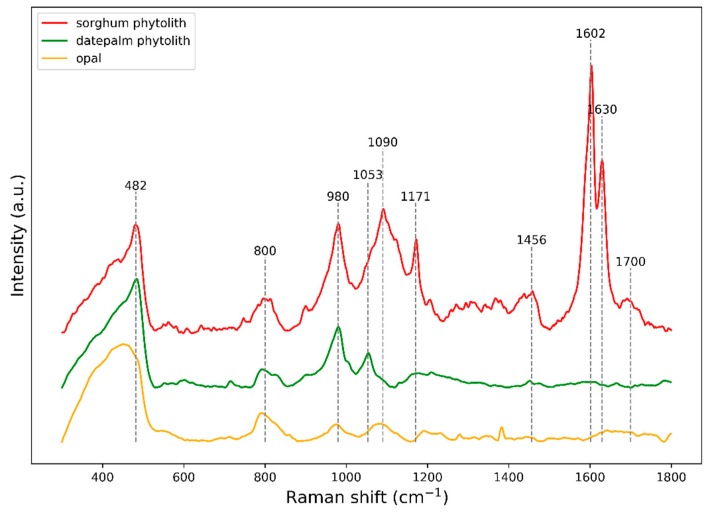
Representative Raman spectra of silica phytoliths isolated from endodermal cells of *Sorghum bicolor* seminal roots (red) and from the root stegmata of *Phoenix dactylifera* (green) [54] (modified). Opal spectrum (yellow) is displayed as a reference. All spectra display two broad and asymmetrical bands in the regions 400–490 and 780–820 cm^−1^ underlining the amorphous nature of the silica.

In an effort to study the specific composition and properties of the two different types of silica phytoliths, the authors analyzed the silicon aggregates by Raman microspectroscopy [41,54] (Figure 3). The studies compared the phytoliths from a date palm (green) and from the root endodermis of the grass, sorghum (red), with an opal spectrum (yellow) as a reference. All spectra displayed two broad and asymmetrical bands (400–490 and 780–820 cm^−1^) underlining the amorphous nature of the silicas, and a notable band (around 98 cm^−1^) indicating Si-OH bonds. The main difference which was found comparing phytolith from the palm and from the sorghum was that the sorghum phytolith spectrum showed a well-resolved signal of entrapped remains in its organic backbone (1171, 1602, and 1630 cm^−1^), and was structurally similar to the composition of endodermal cell walls. The phytolith from the date palm did not contain any organic backbone [54]. Other studies have confirmed that phytoliths isolated from grasses are typically joined with the cell walls, and especially within lignified tissues [41,72,75]. Polysaccharides of the cell wall have also been suggested to interact with Si [78,79]. Mixed-linkage glucans present in the cell walls of Poales and Equisetales might serve as a template for Si polymerization in plants [80]. There are also studies on rice cell wall mutants which show a positive correlation between the content of Si in the biomass and cellulose, hemicelluloses, and lignin in the cell wall [81].

The precipitation and polymerization of Si is the process of so-called biosilicification [28] or biomineralization. The biomineralization process prevails to the above-part plant parts and is still not fully understood [68,72]. There are two contrasting opinions in the scientific literature explaining this phenomenon. According to several studies, Si deposition is a passive process resulting from the condensation of Si, and thus it is directly dependent on the organ transpiration which causes sap dehydration in shoots [22,82,83]. A recent review [84] of this issue indicates that several specific components of the cell walls or cuticular structures affect this process. Another idea inclined to this hypothesis is that Si deposition is an active catalyzed process [72,85,86]. After several studies, it could be biologically induced or controlled [87]. A new insight into the process of silicification produced a recent study where the authors observed the formation of silica aggregates in sorghum roots only in metabolically active endodermal cells, and thus confirmed that this process is active and under tight regulation [69]. They also contributed to the opinions that silicification is not only a passive and metabolically inactive process joined with the plant transpiration. 

## 4. Silicon Transport in Plants

The variation in Si content among plants is the result of Si transport activity by the root system [88]. Early studies proposed three modes of Si uptake by roots: active, passive, or rejective. According to this, plants can be divided into high, intermediate, and low accumulators of Si [89,90]. Experiments with rice mutants show low Si concentration in shoots, and a kinetic study using metabolic inhibitors suggested the presence of Si transporters responsible for Si uptake and xylem loading [90,91]. These results led to the identification of the genes, *Lsi1* and *Lsi2*, coding for membrane proteins responsible for Si transport in roots of rice—a typical Si accumulator species [63,92]. Subsequently, the channel Lsi6, which is homologous to Lsi1, was found in the shoots playing a key role in Si distribution pattern in the leaves [93].

The channels Lsi1 (NIP2-1) and Lsi6 (NIP2-2) are characterized as aquaporin-like proteins and represent members of the nodulin 26-like intrinsic protein III (NIP III) group [63,94]. These proteins function as diffusion facilitators of water and small uncharged solutes across the cell membranes. The Lsi1 is an influx protein responsible for Si uptake by root cells, however, it is also capable of arsenite and boric acid transport across the membrane [95]. The main features of Lsi1-like proteins (and/or proteins of NIP III group) include NPA boxes and ar/R filter, which are also referred to as NPA motifs and GSGR filter, respectively [63,96,97]. The NPA box creates an hourglass-shaped pore required for the transport function, and the GSGR filter acts as a selectivity filter for the transport of solutes such as silicic acid [95,98,99]. The distance between NPA boxes also plays a crucial role in terms of selective transport of Si. It was found that the distance of 108 amino acids between NPA domains is characteristic for Si accumulating species, whereas 109 amino acid distance is typical in non-Si accumulators [96]. According to the molecular data, whether aquaporins contain the mentioned features responsible for Si transport and/or permeability, a different classification of plants in terms of Si uptake was suggested, i.e., accumulator and/or non-accumulator species [100]. However, in contrast to these findings concerning Si specificity of Lsi1-like aquaporin channels, it has been suggested, recently, that Lsi1-like proteins are not specific for silicic acid and may not play a key role in Si transport in plants [101].

In contrast to the passive channel transport of Si, the active transporter Lsi2 is driven by a protonmotive force and plays a role in Si transport out of the cell in roots [92]. This protein shows high homology with arsenite efflux proteins ArsB, which is found in bacteria, as well as archea [92]. The Lsi2 transporter is a member of the putative anion transporter family and the transport mechanism is most likely based on Si(OH)_4_/H^+^ antiport [92,102]. The structure and transport mechanism of Lsi2 is still not well-established and needs further investigation. In the rice genome, there are other four homologs of *Lsi2* and among these homologous genes, *Lsi3* was used for heterologous expression in *Xenopus laevis* oocytes and thus functionally annotated for efflux transport activity [103]. 

The tissue localization of Lsi1 and Lsi2 proteins in roots differ among plant species and among seminal, lateral, and crown roots as well. In rice, the influx Lsi1 channel is located distally and the efflux Lsi2 transporter is found proximally in exodermal and endodermal cells of the seminal, lateral and/or crown roots [63,92,104]. Both Lsi1 and Lsi2 show polar localization and their cooperation is necessary for efficient transport of Si [97,105]. Due to the large aerenchyma in rice roots, Lsi1 and Lsi2 are completely absent in the mesodermal layer. Other species, such as maize, localize Lsi1 in the epidermis and hypodermis of a seminal root, as well as in the epidermis and all cortical cells (except for the endodermis) in lateral roots [106]. The endodermal layer of seminal and lateral roots is occupied by the Lsi2 transporter with a non-polar orientation [102]. The situation is quite different in barley. Here, Lsi1 is polarly situated in the epidermal and all cortical cells, except for the endodermal layer of seminal root and only in the hypodermis of lateral root [107]. The Lsi2 is localized in the endodermal layer exhibiting non-polar orientation. Thus, the Lsi2 transporter may transport silicon from endodermal cells to vascular tissues or back to the root cortex in maize and barley [102]. Localization of wheat Lsi1 channel was shown in transgenic *Arabidopsis* seedlings, where Lsi1 was expressed in all root and shoot tissues with the strongest signal in root cylinder and root hairs [108]. The Lsi6 channels are found in roots tips with polar localization at the distal side of all root cells in rice [93]. In contrast, the maize Lsi6 channel is localized in seminal lateral roots and crown roots without polar orientation [106]. Despite the localization of Lsi6 in roots (which is less abundant), the main localization is in the xylem parenchyma cells of leaf sheaths and blades. In the nodes of rice, Lsi2 and Lsi3 transporters and Lsi6 channels play a role in the intervascular transfer of Si, which is responsible for preferential Si transport and distribution [103]. The *Lsi1* and *Lsi6* transcripts were also found in various tissues of the kernel during its development, however, *Lsi2* was not expressed [109]. In dicots, pumpkin Lsi1 was localized in all root tissues, and interesting to note, with no polar orientation [110], however, when this protein was expressed in rice seedlings, the polarity of pumpkin Lsi1 was observed on the distal side [110]. This may depend on specific factors present in root cells, rather than the protein properties of the transporter [110]. The pumpkin *Lsi2* transcripts were found to be localized in the roots as well as the shoots, however, specific tissue localization of transporter proteins was not examined [95].

Silicon transport proteins in roots, either passive or active, are found in different species throughout the plant kingdom [97] and their number is increasing due to intensive genome sequencing. However, only a limited number of root Lsi1-like and Lsi2-like channels and transporters, respectively, have been functionally annotated. Based on functional characterization, the Lsi1-like proteins are mainly found in monocotyledonous species in the Poaceae family, which includes rice, maize, barley, and wheat [63,106,107,108,111]. Recently, the Lsi1-like channel was functionally demonstrated in *Phoenix dactylifera*, a date palm belonging to the Arecaceae family [54]. In dicotyledonous plants, functional characterization of Lsi1-like channels was initially performed in pumpkin (*Cucurbita moschata*) [110], then later in soybean (*Glycine max*) [112] containing two functional homologs, and recently in cucumber (*Cucumis sativus*) [113]. Current, functional Lsi1-like channels were proven in *Nicotiana tabacum* and *Nicotiana sylvestris* [114,115]. *Nicotiana sylvestris* Lsi1-like channel (NsLsi1) maintains key molecular features (as mentioned above) showing effective Si transport, despite this species being a low Si accumulator [115]. NsLsi1 amino acid sequence contains proline (P) in position 125—a position with highly conserved phenylalanine (F) in other plant species, including Si accumulators [115]. The substitution of these amino acids is responsible for decreased expression of Lsi1 proteins in the cell membrane and thus decreased Si content in tobacco plants. Therefore, the P125 residue may serve as a novel determinant for Si permeability in Lsi1-like protein sequences [115]. In a similar way, it was shown that substitution of proline to leucine at the position of 242 resulted in decreased transport activity of Lsi1 in pumpkin, probably due to improper protein folding [110]. From these data, it appears that not only the main features such as GSGR filter, NPA boxes, and 108 amino acid distance between NPAs contribute to Si transport and/or higher accumulation in plants. 

In addition to the root NIP III aquaporins of flowering plants, NIPs were also found in Pteridophyta, specifically in *Equisetum arvense* with the highest Si content among land plants [116]. These Si channels show homology to the NIP II subgroup with a different STAR filter in comparison to the GSGR filter of NIP III aquaporins. 

The list of experimentally demonstrated activity of Lsi2-like transporters in plants is even shorter and thus offers the opportunity for future experiments. The heterologous expression of *Lsi2* genes in *Xenopus laevis* oocytes was used for OsLsi2 (rice), ZmLsi2 (maize), HvLsi2 (barley) from monocot species and, by contrast, only in pumpkin (CmLsi2) from dicots [92,95,102].

## 5. Effects of Silicon in Plant Roots during Abiotic Stresses

Silicon may be highly deposited in various root tissues and cells in the form of Si-bodies (phytoliths) or associated with the cell wall. The exact question of the role of Si and Si deposits for plant organisms is still a matter of debate. However, relatively broad spectra of experimental works have shown that root silicification might be connected, especially with strengthening the plants against various forms of abiotic and biotic stresses, as well as mechanical hardening of root tissues [117,118].

The deposition of Si in the form of Si phytoliths in the root tissues is a phenomenon known from many plant species. In addition to the suggested role of Si phytoliths in protecting plants against drought, one important role might be in hardening the mechanical resistance of the central cylinder. This was recently indicated on the developmental aspects of silica phytoliths located in stegmata in the root of a date palm [54]. Stegmata are tightly associated with additional mechanical tissue—sclerenchyma—and in this way probably enhance the mechanical protection of the root tissues [54]. Solid Si phytoliths are usually located in older root parts. However, in the case of other plant species, Si associated with the cell wall is deposited in younger root parts as well, where it may function in a contradictory manner. Silica in young primary cell walls interacts with pectins and polyphenols and because of this increases the elasticity of cell walls [119]. The presence of Si in cell walls of tissues that are a part of the elongation and differentiation zone of young root apical segments increased the extensibility of the cell wall [120,121]. Similarly, it was realized that roots treated with Si showed higher plastic and elastic cell wall extension in the elongation zone of maize roots [122]. Therefore, Si is likely responsible for improved root elongation and the enhanced root growth of plants.

It has been shown that Si accumulation and deposition in roots are responsible for better resistance of roots against the toxicity of various heavy metals and toxic elements. Si-Al deposits on the outer epidermis of sorghum root cell walls may be related to the mechanisms responsible for the enhancement of sorghum growth in excess aluminum [123,124,125]. Deposits of Si in the cell wall of the endodermis along thickened Casparian bands were found [126], and the study assumed that the Si deposition is directly related to inhibition of apoplasmic transport into the inner root tissues. Parallel localization of Si and Cd in rice roots was also found [44] and suggested that co-precipitation of these elements in the surroundings of the endodermis related to the physical blockage of apoplasmic transport through root tissues. Similarly, Si deposits were detected in the cell wall of the epidermis, exodermis, endodermis, pericycle, and xylem in maize [127]. The same tissues also represented the sites of Cd and Zn localization. Therefore, they suggested that in those tissues where Si and Cd + Zn are deposited together, a co-precipitation of Si with metals might occur. There is more evidence that cell wall-bound Si might be responsible for the retention of heavy and toxic metals, and thus decreases the metal availability and toxicity. For example, an enhanced ratio of apoplasmically bound Cd and decreased ratio of symplasmically bound Cd due to Si application was observed in the roots of various plant species [50,128,129]. However, it was also observed that the addition of Si did not influence the distribution of Cd within different cell compartments in roots, however, in shoots, significantly more Cd was bound to cell wall fraction [130]. Hard soluble aluminosilicates and/or hydroxyaluminosilicates were formed in cell walls and reduced the content of free Al^3+^ in root symplasm [131]. The greatest amount of Cd is co-localized with hemicellulose fraction of cell walls and suggested that a hemicellulose-bound form of Si with net negative charges is responsible for the inhibition of net Cd uptake in rice cells by a mechanism of (Si-hemicellulose matrix)-Cd complexation and co-deposition [132]. Therefore, these findings clearly demonstrate that the deposition of Si to the root cell walls plays an important role in decreasing metal availability and toxicity, thereby reducing metal stress. A recent review [133] proposed a scheme of the amelioration effects of Si by metal stress. The authors divided the mechanisms into an external part which included growth, changes of pH in media [131], releasing of the chelators, regulation of the metal transporters activities or co-deposition with metals, and an internal part where they counted antioxidants, cell compartmentalization, gene expression modulation, inhibitions of the heavy metal transport into the shoot [44,134], structural alterations or co-precipitations. The importance of the belowground plant parts highlights the fact that it is involved in all of these processes.

There are also findings that document the role of Si deposition to prevent drought stress. An increased number of Si deposits associated with the root endodermis in upland drought resistant rice cultivar was found, and in contrast, the lower density of Si deposits in the endodermis of lowland rice cultivar [135]. The authors suggested that density and deposition of Si phytoliths probably related to the better resistance of upland cultivar to drought conditions [135]. Additionally, cultivars of sorghum that are more drought-resistant contained more Si in root tissues, which prevents water leakage from the central cylinder towards the soil [120]. Therefore, it seems that Si deposition in root tissues, especially around the cell walls of the endo- and exo-dermis, significantly improves water retention and consequently improves the maintenance of plant water balance [135,136,137]. Although it was proposed that endodermal silicification does not support root water retention capability, root silicification might help overcome the stress of drought by decreased inhibition of root growth imposed by desiccation [41]. Since the root is in tight contact with other microorganisms in the soil, the role of plant growth promoting bacteria (PGPB) as well as mycorrhiza, and their interaction with Si and abiotic stress factors should be also considered. This does not seem to be an intensively studied topic, however, some reports indicated that Si applied together with PGPB may help to overcome the drought stress or improve the vigor of plants grown in saline conditions [138,139].

## 6. Effects of Silicon in Plant Roots during Biotic Stresses

Plant roots encounter many pathogen species in their environment. These biotic stressors cause retardation of growth, yield production, and shortening of lifespan in hosts. According to the experimental studies, Si in plant roots can alleviate the stress after bacterial [140,141,142], fungal [143,144,145], insect [146,147], nematode [148,149], and plant [150,151] attack, but also stress from auto-toxicity of the same plant [152]. The level of its beneficial effect is Si concentration dependent. Typically, when the uptake and accumulation of silicon by plant roots is higher, the negative effect of root pathogen attack is lower. In general, resistant plant cultivars accumulate larger amounts of silicon in the roots with a more prominent and faster alleviating effect than the sensitive cultivars [148,153]. Plants are able to improve silicon acquisition via interaction with microorganisms in the rhizosphere by formation of mycorrhizas [153,154,155]. However, the total Si concentration in the plant during alleviation of biotic stress is important, since its localization inside a root is even more crucial. A comparison of plant species with different index of symplasmic/apoplasmic silicon content has shown that plants with higher symplasmic silicon content are more resistant to fungal pathogen [154].

The alleviation effects of Si amendment in plants during a biotic pathogen attack is usually limited to some degree. There are only a few known plant root–biotic stressor interactions where the presence of silicon reduces the negative effects of stress completely [140] or even overcomes the treated plant biomass production compared to non-stress conditions [156]. In most of the other cases, silicon alleviates the negative effects to some degree, ranging between 10% and 80% (e.g., in [157,158,159,160,161]). For example, in silicon-treated tomato plants, the bacterial wilt incidence, which is caused by *Ralstonia solanacearum*, was reduced in roots by 100% and by 38% in resistant and moderately resistant cultivar, respectively [140]. Those ameliorative effects are caused at least partially by changing soil microbial composition through an increase in the number of beneficial bacteria and actinomycetes. They improved the soil urease and soil acid phosphatase activity and caused a decrease in the numbers of wilt causing bacteria in both the soil [162] and the plants [156]. It has been shown that Si-mediated enhanced resistance and tolerance in tomato to *R. solanacearum* is associated also with differential expression of genes involved in pathogen-associated molecular pattern-triggered immunity via modification of signaling pathways and energy metabolism [141,142]. Coconut palms (*Cocos nucifera*) infected by phytoplasma that causes root wilt were able to influence the rhizosphere via secretion of compounds that promote beneficial microorganisms, such as N_2_-fixers, silicate solubilizers, and actinomycetes. They eliminated the proportion of pathogenic bacteria and increased acquisition of Si by plants which manifest in improved growth and production of palms [162].

There are many studies which prove that Si-amendment can significantly reduce disease incidence and severity in the roots of plants infected by fungi [143,144,145,158,163,164,165,166,167]. Cucumber (*Cucumis sativus*) and banana (*Musa acuminata*) plants fertilized by Si and threatened by *Pythium ultimum* and *Fusarium oxysporum*, respectively, increased chitinase and beta-1,3-glucanase activity and were able to degrade the body of a pathogen [144,167]. Plants also activated peroxidases, phenylalanine ammonia lyases, and polyphenol oxidases [144,167], resulting in increased production of total soluble phenolics and lignin-thioglycolic acid derivates [167], which play a role in cell wall improvement and plant immunity. Si-amended roots produced more glycosidically bound phenolics and aglycones with proven fungistatic activity against several fungal pathogens [144]. In Si-treated cucumbers (*Cucumis sativus*) infected by *Phytophthora melonis*, the roots increased the activities of catalase and ascorbate peroxidase. These activities of the antioxidant enzymes improved the plant resistance to oxidative stress and ameliorated plant growth inhibition caused by the pathogen [165]. Silicon was involved in reduction of root necrosis, decay, and mortality [143,158] and in improvement of root growth and biomass production due to balancing in micronutrient uptake and translocation inside the cucumber plants [165]. Roots also play an important role in delaying the initial infection and movement of the fungal pathogen from roots to the aboveground part of plants [166]. The invasion and distribution of necrotrophic fungus *Alternaria alternata* through the root tissues of sorghum (*Sorghum bicolor*) was restricted at the level of the exodermis, probably due to silicon mediated modifications of cell walls via deposition of phenolics and suberin [145]. However, it needs to be noted that plants unable to take up silicon sufficiently, such as poinsettias, cannot inhibit the onset of symptoms and severity of fungal attack [168].

Silicon amendment can alleviate the effects of an animal attack as well. It has been shown that after infestion by the root-knot nematodes *Meloidogyne exigua* and *Meloidogyne paranaensis*, the roots of the Si-amended *Coffea arabica* plant significantly decreased the nematode gall and egg number, as well as reproductive capacity [148,169]. The likely reason was the larger production of lignin-thioglycolic acid derivates and increased activity of peroxidases, polyphenoloxidases, and phenylalanine ammonia lyase, which were even more prominent in cultivars that accumulate higher amounts of Si [148]. However, this tendency is not generally valid for all nematode species and all plant hosts. In the case of the banana Prata-Ana, the decreased egg and gall mass/root and number of second juveniles of *Meloidogyne javanica* per defined soil volume were not observed in silicon treated plants [170] and there were no ameliorative effects of Si in maize plants infested by this pathogen. On the other hand, significant positive effects of Si were observed in soybean, common bean, and rice [161]. After Si-treatment, the genes were overexpressed in more resistant cultivars of rice, playing a role in defense via the ethylene signaling pathway, and roots deposited more callose and phenolic compounds compared to Si-non-treated plants [149].

An increased amount of silicon can slow down the infestation by insects as well. Silicon fertilizer application into the soil and its uptake into the root system of *Brachiara brizantha* and sugarcane resulted in the reduction of nymphal number of brown root stinkbug and increased nymphal mortality, as well as decreased longevity of imagos of hemipteroid spittlebug *Mahanarva fimbriolata* [146,171]. The presence of arbuscular mycorrhiza can increase the Si ameliorative effects during insect attack in two distinct ways: (1) by increasing the silicon acquisition by plants and improving their fitness, and (2) by priming insect immunity, due to presence of microorganisms, which costs the predator more energy and results in its growth reduction [172]. The accumulated silicon can protect even the adventitious roots in the aboveground part of plants against insect pathogen, where, for example, the root primordia on the stalk of sugarcane were less consumed after infestation by lepidopteran pyralid borer *Eldana saccharina*, resulting in a decrease of the borer growth rate and survival [173]. The higher the Si content in the stalk epidermis covering the aerial nodal adventitious roots of sugarcane, the greater was the inhibition effect on the herbivore [147]. It was hypothesized that the borer inhibition can be caused by its mandibular wear during consumption of silicon-containing hard plant material, and thus continually lowering the amount of plant damage by the herbivore, however, this was not confirmed [174]. When the roots are not able to transport silicon into the aboveground parts of plants, such as the case of pepper (*Capsicum annum*), the inhibition effects on feeding and reproduction of pathogenic insect predator chilli thrips (*Scirtothrips dorsalis*) are not present [175].

Plants can be infested even by parasitic plants, and host plant roots may play a crucial role in the suppression of such biotic stress. The roots of sunflowers (*Helianthus annuus*) that fed well on nutrients and beneficial elements, including silicon, were able to withstand better the attack of the root parasite angiosperm broomrape (*Orobanche cumana*). The result was a decrease of parasite attachments to the host, inhibition of parasite stem development in the substrate, and a higher level of broomrape necrosis [150]. The resistant cultivar of tomato plants suffering from Egyptian broomrape (*Phelipanche aegyptiaca*) attack reduced damage severity by increasing root peroxidase and catalase activity [151]. A positive effect of silicon has also been demonstrated in the resistance of host plants when attacked by the parasitic angiosperm plant—dodder (*Cuscuta europaea*) [176]. The parasite was not able to wrap around and penetrate tobacco (*Nicotiana benthamiana*) plants by haustoria after foliar application of Si.

Based on the results mentioned above, the reduction of disease incidence and severity in plants by Si is caused on several levels:

(1) in plants by modification of gene expression and metabolome resulting in
  a: limitation of pathogens spreading via deposition of phenolics and suberin into the cell walls, and thus changed their composition and structure,
  b: reduction of oxidative stress via synthesis of enzymes decreasing the amounts of reactive oxygen species,
  c: decreased wounding caused by pathogen via increased production and directed secretion of plant enzymes degrading pathogens’ bodies, toxic and allelopathic substances,
  d: decreased secretion of effectors involved in compatible plant–pathogen interaction mimicking incompatible interaction and thus causing failure in pathogen attack,
  e: improvement of nutrient uptake and distribution via improved interaction with microorganisms involved in arbuscular mycorrhizas,
  f: increased biomass production and growth of a plant and in retardation of plant death via induction of pathways releasing energy and substances from its own resources.
(2) in environment by changing the biotic and abiotic factors via plant secreted substances that promote reproduction of beneficial bacteria, actinomycetes, N_2_-fixing organisms, and silicate solubilizing microorganisms, and thus decreased the number of pathogens.
(3) in pathogens by retardation of its reproduction speed, survival, lifespan, fertility, viability, and attack caused by a decreased amount of nutritious plant food and increased expenses to eliminate intoxication by allelopathic substances.

## 7. Conclusions

The knowledge about Si uptake, translocation, deposits, and Si role in plants has been accumulated in existing literature for more than 150 years. The increasing interest in recent years has been stimulated mostly by the discovery of Si channels and transporters in roots (in [59] and subsequent literature), and also by the growing evidence regarding the role of Si in plant resistance to biotic and abiotic stresses. There are two major places of Si deposits in roots. One is in the endodermal tissues where two basic forms can be characterized: either in the form of impregnation of the endodermal cell walls, or in the form of Si aggregates in the endodermal walls known in the Andropogonae (Poales) tribe. The other, less known location of Si deposition is represented by Si phytoliths in specific stegmata cells associated with sclerified tissues and sclerenchyma fibers that are known in several groups of plants. There are several places of Si deposition in roots, which are even less known, less studied, and less understood, such as impregnation of xylem vascular tissues by Si and intercellular Si deposits. In all these cases, a clear explanation of the role of these deposits is either lacking or contradictory. Apoplasmic movement of Si in intercellular spaces and cell walls with specific composition and different porosity is not yet clear and it has been recommended recently as a perspective research topic [101]. Other less-known mechanisms of root silicification include Si interaction with cell metabolism, transport mechanism of Lsi2 proteins, and Si accumulation in the root exodermis as well. The function of Si deposits in roots in reaction to biotic and abiotic stresses is much less known than it is in the aboveground parts of the plant. Changing environmental conditions, such as increasing air temperature or increasing concentration of CO_2_ as a consequence of climatic changes, should also be taken into consideration when Si fertilization is applied in agronomy praxis. The evidence of its positive effects is increasing from various plant species, however, more research will be required to explain and understand it.
